# A TECHNOLOGY SUPPORTED FALL PREVENTION INTERVENTION FOR OLDER ADULTS WITH MILD COGNITIVE IMPAIRMENT

**DOI:** 10.1093/geroni/igad104.3782

**Published:** 2023-12-21

**Authors:** George Demiris, Nancy Hodgson, Therese Richmond, Marjorie Skubic, Justine Sefcik, Sean Harrison

**Affiliations:** University of Pennsylvania, Philadelphia, Pennsylvania, United States; University of Pennsylvania, Philadelphia, Pennsylvania, United States; University of Pennsylvania, Philadelphia, Pennsylvania, United States; University of Missouri, Columbia, Missouri, United States; Drexel University College of Nursing and Health Professions, Philadelphia, Pennsylvania, United States; University of Pennsylvania, Philadelphia, Pennsylvania, United States

## Abstract

Falls and fall-related injuries are significant public health issues for adults 65 years of age and older. Over a third of older adults (OA) fall each year and 10-20% of falls result in serious injuries. Cognitive impairment has been identified as a leading risk factor with over 60% of OA living with mild cognitive impairment (MCI) falling annually. Housing conditions are also a risk factor for falls. We have developed Sense4Safety, a technology-enhanced intervention that includes multiple components: 1) in-home assessment and action; 2) education and regular communication with a coach; 3) passive monitoring facilitated by depth sensors in the home; and 4) alerts. The goal of this study was to access the feasibility of Sense4Safety and its acceptability. We recruited 10 low-income OA with MCI; each participant received the intervention for 3 months. All participants completed all study procedures. The Short Physical Performance Battery (SPPB) scores which provide an objective assessment of lower extremity functioning based on clinical experts’ observation showed high correlation to the sensor system assessment (r=0.94, p< 0.01). Two fall alerts were generated during the study period that triggered a timely response. Participants and their family members found the intervention to provide peace of mind and empower OA to proactively address changes in fall risk through exercise and home modifications. The combination of passive sensing and personalized coaching offers an innovative ecological approach to continuously assess functional mobility and gait in the home and captures changes that provide an early warning for fall risk.

